# Lost in Digitisation: The Film Reel and the Craft of Women’s Home Movie Making

**DOI:** 10.1080/10509208.2025.2518361

**Published:** 2025-06-16

**Authors:** Carolann Madden, Sarah Arnold, Kasandra O’Connell

## Introduction

This article extends from two amateur film projects concerned with examining women’s amateur filmmaking and the development of metadata schemas and archival practices that could foreground women’s contributions to amateur filmmaking. Both “Women in Focus” and “Empowering Archivists” were UK-Ireland projects that facilitated collaborations between archivists, researchers and community groups to collectively think of ways to better represent, value and preserve amateur films made by women. One of the findings of these projects has been the understanding that women’s amateur filmmaking necessitated looking beyond the film content to the material objects of the films themselves.

Examination of the 8 mm films that formed the Margaret Currivan Collection, held at the Irish Film Institute’s Irish Film Archive, revealed the filmmaker’s extensive editing and splicing, along with the inclusion of heavily crafted effects like fades and title cards. All of this told a story about Margaret Currivan as filmmaker, which complemented the story of Currivan as mother, wife and homemaker as revealed by the content of the films. Importantly, the digitised versions of the films also obscured much of this detail because we could not see how these effects were put into practice, only the end result. This observation has led to the current article that: examines how amateur films have typically been understood as content rather than form; considers how women’s filmmaking is “at risk” because women’s amateur film content itself is undervalued; and argues that the trend towards digitisation as preservation should not occur at the neglect of material film object.

Throughout this article, we examine existing trends in the scholarship of amateur film that, following Ryan Shand’s (2008) assessment of the literature, emphasise modes that can be defined as domestic (home movie), oppositional (experimental, avant-garde), and evidential (documentary). While taxonomies of amateur film provide useful lenses through which to theorise and give meaning to amateur films, these modes often prioritise content over form. Fewer studies attend to the practice of amateur filmmaking, especially that of women, nor the materiality of amateur film. The trend towards digitisation in archives means that there may be less incentive for researchers to look beyond content towards the film material. Indeed, scholars like Giovanna Fossati (2018) problematise the adoption of digitisation practices and the implications for the how amateur films are accessed, restored, and understood.

Our intervention, therefore, is twofold: we propose that attending to amateur film not only in terms of content but in terms of authorship and materiality is hugely important for amateur film studies; and we further propose that such an intervention creates opportunities to challenge value judgements that would see “amateur” as a mark of inferiority. This conflation of amateur film content with the “ordinary” and the “domestic” has particularly obscured the craft and creativity of women’s amateur films. Our case study of the Margaret Currivan Collection demonstrates how a focus on material films rather than digitised films reveals the filmmaker’s extensive filmmaking craft, something not evident in digitised films nor traditional film metadata. Over the coming pages, we map the trends and absences in the literature in regards to “amateur”, “film” and “women’s filmmaking” before explaining the processes through which we accessed and examined the Margaret Currivan Collection. We then follow with an exploration of the various filmmaking techniques and skills deployed by Currivan in the production of her home movies, thereby calling attention to the importance of the physical films to the recognition of Currivan’s authorship and craft.

### A Certain Tendency in Amateur Film Scholarship

Amateur film scholarship is dominated by a number of trends, described by Shand as the domestic, the oppositional and the evidential (2008). Shand points to the work of scholars such as Richard Chalfen to explain the domestic tradition, in which Chalfen sees amateur film as serving a particular domestic and social utility of documenting the family and capturing spontaneous moments, and less as aesthetically sophisticated craft (2008, 40). Other scholars have expanded on this domestic tradition, often associated with the home movie in which the filmmaker becomes a curator of the family by recording events and rituals (Odin 2014). Chuck Kleinhans, for example, notes how family history is coordinated quite often by the wife and mother of the family: “Whether through scrapbooks, photo albums, or home movies and tapes, it seems like women are often the historians of domestic space and activity” (1986, 34). Home movie makers are also considered pseudo-ethnographers who capture the “persons, objects, and events” that are represented (Mörner 2011, 29). Even for those scholars interested in home movies outside of the home – for example, travelogues, holiday films, visits to non-domestic sites – the tendency is to see the content as evidence of a social history. Home movies describe the emergence of increasing social mobility, consumer culture, globalisation (Erens 1986; Norris Nicholson 2004; Kerry 2014).

The oppositional tradition, to borrow Shand’s phrase, sees amateur film as subversive and as a counter to dominant commercial cinema (2008 46). Such amateur films are valued because of their difference from commercial film. Indeed scholars (ourselves included) become very excited when they discover amateur films that “transcend” what is expected from the mode. In his review of the 1997 FIAF Symposium “Out of the Attic: Archiving Amateur Film” Jan-Christopher Horak states that “one of the most interesting revelations about amateur films was that such films could also be ‘art’” (1998, 52). He describes some of the surprisingly artistic amateur films screened at the symposium, and uses terms like “alternative avant-garde” to describe them. Various textual analyses of amateur film identify exceptional practices and seek to use the language and terminology of the high and oppositional arts to understand the aesthetics of amateur film (Zimmermann 1986, 1996; Ruoff 1991; Mariani 2018). Equally, those who examine amateur film magazines and cine club practices describe the formal practices of amateur filmmakers, but often in relation to films’ narration and story (Cuzner 2009; Vinogradova 2012; Shand 2014; Tepperman 2014). Maija Howe’s (2014) study of amateur film zines examines how amateur filmmakers were guided towards creating more crafted and formally controlled films.

Shand, finally, points to a final tradition in amateur filmmaking – the evidential tradition – which refers to a trend in which archivists value amateur film because of the visual evidence it provides on place, people and events. Shand (2008) also notes how certain amateur film practices hold more value because of the content and production context. Home movies might be valued because of their evidential and social utility, but he says that this risks obscuring and undervaluing other forms of amateur film that do not easily fall into existing amateur film schemas. Beyond Shand’s three traditions, there are other studies that are concerned with the materiality of amateur film, especially in terms of film gauge (Salazkina and Fibla-Gutierrez 2018; Tepperman 2018). Such studies are concerned with gauge and medium inasmuch as film gauges facilitated certain types of practice and, in some cases, nurtured shadow film industries in some nations where no mainstream industries existed or where commercial cinema culture was difficult to access. These various approaches to amateur film are crucial in developing a comprehensive methodology for and mapping of the breadth of amateur film practice.

Somewhat less attended to in studies of amateur film, however, is the material production of the amateur film by its filmmakers, in other words, the craft of amateur filmmaking. Indeed, much of what is assumed about amateur film is that it is poorly crafted, therefore, unworthy of attention. While some amateur film was produced with little intent towards the production of a crafted material object, the wealth of cine club films, amateur film festivals and the many festival entries documented in places like *Amateur Cine World* evidence the extent to which the films were, indeed, crafted. There is some scholarship that addresses the oeuvre of particular amateur filmmakers and that adopts auteurist approaches to amateur film, but fewer still consider the “making” of amateur film by its filmmakers. How did amateur filmmakers work with film? What did they do with and to the rolls of film they had processed? How did they construct a narrative? The answers are sometimes found in the film reels themselves, where traces of a filmmaker’s practice can be seen in the edits, splices, sound strips, effects that make up the material object.

### Digitisation is Not Preservation

These physical characteristics and manipulations of analogue film strips and reels are easily overlooked in the digitised versions. It has long been recognised that it is archivists who most frequently interact with material film (Streible 2013; Shand 2014; Fossati 2018; Antoniazzi 2021; Cherchi Usai 2010, 2021), and unless academics and researchers examine the physical reels for a specific purpose, then this remains the case. The archival setting emphasises the importance of the materiality of film, but also highlights the distinction between digital and analogue formats. For example, even when an analogue film is digitised, many archives still attempt to preserve the physical reels. In this instance, the analogue film is viewed as artefact. In her seminal text on the transition from analogue to digital filmmaking, *From Grain to Pixel: The Archival Life of Film in Transition,* Fossati examines how this transition affects not only filmmaking but also film archiving. Fossati argues that “in a theory of archival practice the film as artefact, in its different possible meanings, is central” (149). In terms of film digitisation, we follow the lead of Brennen and Kreiss, who consider digitisation to be “the material process of converting individual analogue streams of information into digital bits” (Brennen and Kreiss 2014).

In the early stages of widespread film digitisation in the 1990s, there was a belief that this new practice would not only offer more democratic access to films but would also aid in long-term preservation of both more volatile and more stable analogue film formats. In reality, as Antoniazzi points out, the issue evolved to be more complicated with digitisation debates largely falling into one of two camps: enthusiastic scholars envisioning an exciting digital future for film and film preservation and those, like Cherchi Usai (2021), who warned of a “digital dark age” (Antoniazzi 2021, 196). Cherchi Usai’s concern is particularly related to archives. He worries that the ease of digital reproduction may lead to the view that preserving analogue film prints is unnecessary. Similarly, given the general acceptance that film decays, there is a related concern that digitisation might be perceived as a way to avoid the challenges of material preservation altogether (Cherchi Usai 2021, 251–252). While many film and mixed-media archives advocate for the preservation of the material film, they often have to challenge the view that digital copies should be preservation enough.

The argument can be made that film should be treated no differently than any other artform wherein a digital representation of an original would not be taken as anything other than a reproduction. For example, a photograph of a painting vs. the painting itself. Fossati demonstrates how film restoration is often treated differently than other artforms. She points out that, while there are lively academic debates when a famous painting undergoes restoration, film’s reproducibility fosters, at times even encourages, the disregard of restoration proper. For the most part, film restorations are left entirely to the restorer with, as Fossati reminds us, little demand for documentation of the process afterward, as well as little engagement with academics and scholars throughout (2018, 149–150). And the question remains, what are these restored versions? And what, if anything, is lost as films are digitised and new versions made over and over, issued and reissued? One potential effect is the perception that the original film is surplus to requirement when there is a digital proxy, with the proxy eventually replacing the original.

Scholars like Streible go so far as to argue that we need to reconsider using the word “film” to mean both digital and analogue formats. While recognising that it is not necessarily incorrect to refer to both forms as “films”, he states that “if we forget to specify what photochemical film was, we stand to lose important historical knowledge and awareness” (2013, 229). Streible prefers “moving image” as the term that might encompass all formats of film and understands the rise in the use of “moving image” as coming out of and speaking to the ways in which archives interact with the materiality of film (2013, 229–230). Indeed, for Streible, the exchange between archivists and academics has illuminated the need for a more applicable term, as well as a deeper appreciation for the differences between digital and physical film formats.

### Are Women Amateur Filmmakers or Home Movie Makers?

Much like material film has been overlooked, women’s amateur filmmaking has until recent years been relatively underrepresented in amateur film scholarship. Further, women’s cultural production has often been essentialised or particularised, as though women’s perspective necessarily bears the traces of their gender. Patricia R. Zimmerman’s article “Geographies of desire”, for example, compares a male and a female travelogue filmmaker, finding the woman’s perspective more intimate, more proximate to the position of the “Other” that she records. For Zimmerman (1996, 97), woman’s amateur films are about relationships and not subjects. Katherine Brickell and Bradley Garrett (2013, 4), likewise, essentialise women’s amateur film practice, noting how the amateur films of mountaineer Eileen Healy record scenes that are more domestic and feminine, for example of women cutting each other’s hair. Kleinhans’ (1986) study of his aunt’s amateur films also defines them in relation to family and domestic space, concerned with the content more than the form. Although he aims to recuperate the film practice of his aunt, he also refers to it as hobbyist and pays less attention to the craft evident in the films (e.g. soundtracks, editing, etc.).

Another body of literature recognises women’s amateur filmmaking as accomplished and as technically and aesthetically sophisticated. This literature explores women’s collaborations and contributions within amateur cine clubs and filmmaking groups, presenting such films as evidence for women’s filmmaking abilities and interest. Keith Johnston (2025), for example, examines the amateur filmmaking magazines *Amateur Cine World* and *Movie Maker* for evidence of women working in amateur film clubs. Although he finds such magazines “patriarchal”, Johnston notes that as the decades advanced, magazines gave more attention to women as serious and accomplished filmmakers. Elsewhere, Paul Frith and Keith Johnston (2020) use the Institute of Amateur Cinematographers collections to identify the range of amateur film work undertaken by women. Annamaria Motrescu-Mayes and Heather Norris Nicholson’s (2018) *British Women Amateur Filmmakers* identifies 40 female amateur filmmakers working across a range of genres and undertaking a variety of practices. They argue that there was no overall homogenous “women’s practice”. By identifying the formal practice and craft of these amateur filmmakers, they advocate for recognising women’s amateur filmmaking as a practice.

Kasandra O’Connell, likewise, draws attention to the technique and aesthetics of women amateur filmmakers in the Irish Film Archive and discusses the use of production techniques such as titles, credits and editing within the films that form part of the Margaret Currivan Collection, discussed in this article (2021). O’Connell points to the biases in archival practice that result in inattention to women’s amateur filmmaking and the associated assumptions that it lacks craft and production quality. O’Connell highlights practices that contribute to the neglect of women’s amateur filmmaking, namely, the lack of gender fields in film metadata standards. Scholars, ranging from Sarah Hill and Keith Johnston, and Sarah Arnold and Carolann Madden (2024), have addressed how metadata schemas can contribute to the invisibilisation of women’s amateur filmmaking in the archives. They advocate for metadata standards and practices that can improve access to women’s amateur filmmaking. Charles Tepperman emphasises the value of gendered metadata in projects such as the Amateur Movie Database (2020a, 2020b), whereby the collection of gender metadata revealed a higher percentage of female amateur filmmakers than male. Collectively, these authors demonstrate the role of archival metadata in the uncovering of women’s amateur filmmaking.

Studies of women’s amateur filmmaking often perceive women’s production as domestic and feminine, contributing to its undervaluing. Women’s work receives greater recognition when the production context is more “valued”, such as women’s participation in cine clubs and in more formalised amateur filmmaking settings. The undervaluing of women’s amateur filmmaking is also related to existing metadata standards adopted by archives which may obscure women who did not adopt professional roles and titles. Finally, scholarly attention to women’s production and craft practices remains limited. This article intends to address this gap by focusing on what the material film reveals about amateur filmmaking as well as how this turn to the films circumvents the typical association of amateur films with “poor” filmmaking (Mörner 2011).

## Methods

Our article adopts a qualitative approach to the study of amateur filmmaking and draws from archival research and film studies methodologies through a case study of an individual film collection held at the Irish Film Archive: The Margaret Currivan Collection. This research formed part of the activities undertaken during the Women in Focus project, funded by the AHRC and IRC/Taighde Éireann Digital Humanities award, as well as the UKRI-funded Empowering Archivists project, in which scholars and archivists from the UK and Ireland collaborated on studies of women’s amateur filmmaking. We adopted a feminist historiographical approach to the study of women’s amateur films and filmmaking by which we examined and interrogated the available sources on women’s media histories but also recognised that these histories are often missing, obscured, neglected or unattended to. Archival research was central to the study as we drew from materials contained within the Irish Film Archive and which had been donated by the Currivan family including: the film reels and their packaging; digital reproductions of the films; the film content; film records; technical records, collection level summaries; and donor agreements. However, while the archive was an obvious and crucial source of information about Currivan and her films, we recognise that the archive is only ever a partial history and one that is impacted by biases. After all, archives are selective. They can make value judgements and form hierarchies of cultural and creative production (Noordegraaf 2015). Equally, audiovisual archives, like the audiovisual industries more generally, have not been kind to the legacy and histories of women wherein work by women tends to be underrepresented and undervalued, especially amateur film (O’Connell 2021; Johnston and Hill 2020). Audiovisual metadata schemas also often speak to professional and commercial film rather than amateur film and, therefore, may not represent amateur films effectively. In our study, for example, we note that data on the technical and practical elements of the films was not represented in the film records, thereby concealing some of Currivan’s authorship.

Our study of Currivan’s work also necessitated textual analysis of the physical films and we viewed all 24 8 mm films in her collection. We examined the films’ content and representations, and looked for evidence of Currivan’s interests and concerns. We noted topics, people and places that appeared. It is important to note, however, that we examined the films in both analogue and digital form, since different authorships were revealed within each. Digital films undoubtedly proved easier to access and assess and could be easily viewed repeatedly. The analogue films, on the other hand, were more difficult to view. We had to view the films on-site and the manual viewer was clunky to operate, the moving images small. However, viewing the films in this way allowed us to see Currivan’s craft at work. The 8 mm reels revealed the edits, splices, fades, all of the work of creating the films. We combined our textual analysis, therefore, with analysis of the material object. We cross-referenced the information we gathered from the film reels and analogue film viewing with that of the digital films and of the metadata and film records. Ultimately, we found that the film reels themselves are of huge importance to the understanding and study of amateur film and it is especially important not to rely on digitisation as the proxy for celluloid amateur film formats in particular. Examination of the film reels themselves helped us to gain further understanding of women’s film and home movie making. In the following sections we expand upon these findings and offer some recommendations for the preservation of amateur film and the importance of this particularly for the recognition of women’s amateur filmmaking.

### The Margaret Currivan Collection

Margaret Currivan (1923–1985, née Meagher) and her husband P.J. (Patrick Joseph) (1923–1981) were owners of Currivan’s Photographic Service and Pharmacy, Keeper Rd., Crumlin, Co. Dublin. Margaret was an avid amateur filmmaker and shot most of the scenes in the Margaret Currivan Collection. While P.J. was also an amateur filmmaker, his films mainly focused on his hobby of steam engines and railway-related activities. His films were given to the Irish Railway Record Society (an organisation he chaired) prior to the Irish Film Institute receiving the remainder of the collection and there is a room dedicated to him in Heuston Station, Dublin, the location of the IRSS library. The films shot by Margaret were donated to the IFI Irish Film Archive by her daughter, Helen Redden in July 2007 who felt her mother’s output was worthy of the recognition and preservation already afforded her to her father’s work.

Margaret’s films were made over a 10 year period from the mid-1950s to the mid-1960s, and consist of 24 rolls of 8 mm film. The films predominantly feature the three Currivan children Patrick (1948–2001) Dan (1953), Helen (1955–2008), family holidays in Butlins, Rush and Athlone, birthdays and holidays, and school and leisure activities. Of particular note are two mini-documentaries which are both charming and technically accomplished. The first, *A Day to Remember,* documents daughter Helen’s First Holy Communion, but is told from the child’s point of view, giving it a poignancy rarely seen in amateur work of this period. Similarly, *Up the Canal* depicts an old man reflecting on a changing Dublin, with his thoughts given life in the voiceover. The resulting film is melancholy and evocative, permeated with a sense of regret and nostalgia. Margaret was an ambitious and creative filmmaker who endeavoured to create personal films, not only giving expression to her own creative ambitions but also seeking to explore the experiences of her relatives. Her use of handmade titles and intertitles, careful editing and scripted voiceovers further enhances the sense of intimacy and authorship within her productions. It is precisely the crafted aspect of these effects that might be missed when viewing digital copies of the films. Our concern, throughout the remainder of this article, is to document how we can understand amateur films through the films themselves and what they reveal about the amateur filmmakers’ craft and concomitantly to advocate for the importance of retaining emphasis on material film.

### The Materiality of the Currivan Films

All of the films in the Margaret Currivan Collection display some element of physical manipulation of the reels. This occurs most frequently in form of splices, but also occasionally in the form of things like fades or combined magnetic strip sound and voiceover. Gwenda Young makes note of the many techniques of which Margaret Currivan is most fond, for example, establishing shots, extreme closeups, semi-abstract shots and occasional voiceover, and acknowledges that Currivan exhibits a “clear understanding of narrative construction” (Young 2014, 90). While these techniques are clearly visible in the digitised films, the true weight of their use is best understood in the context of the physical reels themselves. It is sometimes easy to forget in the 21^st^ century that editing of this nature had to be done by hand, typically in a dark room, hunched over a manual viewer lit with a small lightbulb, along with a manual splicer and either rolls of splicing tape or a bottle of film cement and a tiny brush. In an interview conducted by Young with Margaret Currivan’s son, Dan Currivan, he states that his mother “did all the editing herself” as far as he remembers and that she showed him how she did it with a manual viewer “cutting and splicing as she went” (90). Splicing itself is not necessarily difficult if you are not concerned with storytelling or continuity, but splicing with intent for creating a narrative is labour intensive and time-consuming. This is the kind of splicing that Margaret Currivan does most often.

Even in the most “conventional” of her home movies, Currivan’s splicing is done with particular intention, as she often creates narrative arcs and employs spliced-in title cards and intertitles. In fact, all but three of the 24 reels in her collection utilise title cards of some kind in this way. You might expect to find fewer physical manipulations on the earlier films, and that does seem to be the case; however, even the earliest reel, Reel 18 from 1954, utilises titles and intertitles. Reel 18 is comprised of four separate family events and outings with separate titles for each. While the fourth event, Easter, is fairly typical home movie fare, the first three events are not really events in the sense of holidays or family trips, and yet, they are still marked by their own titles. The first, children playing by the water in the Irish town Athlone, is titled “Paddy and Lanes in Athlone August 1954” on a sign with yellow letters cut out and glued on a black background and shot propped up against the wall. The next, a brief section of the very young Currivan children playing in their back garden, is accompanied by a similar sign, positioned in the same way in the same room, that reads “Paddy-Dan Michael Nov. 1954”. While these two titles function by way of naming what the viewer is about to see, the third title functions more so as an intertitle announcing, “At last he found his feet” typed onto a white background and spliced in between a shot of Dan as a baby sitting in the garden playing with a bucket and Dan later in the same garden toddling around. The decision to splice in titles and intertitles, particularly for comedic effect, evidence the craft undertaken to create family stories.

On the digitised versions of the films, it is easy to pass over these title cards, regarding them as merely markers between events, but when considering the effort that goes into inserting them, they take on a different meaning. It is worth emphasising this effort here. Currivan is, in effect, making these cards by hand, some of which become very elaborate as the years goes on. The fact that some of the film reels date a year or two after the filmed events suggests that she was returning to the developed films at a later point and creating intertitles to identify the activities captured and to add a humorous tone to the films. Equally undetectable in the digitised films is the method of splicing used by Currivan. There are two methods. With tape splicing, the filmmaker can cut the film anywhere, typically using a film splicer, and simply connect the two sections of film by taping them together, often with the splicer’s built-in roll of splicing tape. The other method is cement splicing, which is more challenging. This requires the filmmaker, working again with two sections of film, to carefully scrape the emulsion off of a frame from one section of the film, then glue the two sections together, resulting in the loss of a frame. Cement splicing requires more precision as cement splices cannot be taken apart once dry and redoing them means cutting out additional frames. Currivan can be seen using both methods in her collection.

Currivan’s dedication to not only the documentation of events but also the physical craft of editing and filmmaking can be seen particularly in reels that make extensive use of titles and intertitles. Reel 10, for example, has thirteen titles and intertitles, a few of which are also animated. The second title card in the reel displays a jumble of letters on a black background that are eventually arranged letter by letter to read, “Feb. 1958—Lessons”. What follows is a vignette of about one minute long that presents the Currivan children doing their homework through a sequence of mid-range shots of them with their books at the table, as well as close-ups of their hands practicing writing in both English and Irish and doing sums. A few minutes later on the same reel, there is another minute-long sequence of the children playing with instruments and dancing around prefaced by the title, “Music Hath Charms”. This sequence also features a brief animation of music notes moving against a blue background spliced in, and an intertitle that reads “Far too frivolous for me” spliced in before a shot of a child watching the others from a bed. While these short sequences do feature the children, they appear to be about more than documenting family life. They exhibit Currivan’s innate creativity and technical skill being applied to even the everyday and ordinary moments of family life. Looking at the original reels shows Margaret Currivan going beyond simply documenting her family to carefully manipulating the film reels.

These manipulations and 8 mm editing techniques are also easy to miss within other technically and formally complex films made by Currivan. For example, *Up the Canal* (1966), her documentary-style short reflecting on Dublin’s Royal Canal, exhibits hand-crafted effects that one would barely notice in the digitised version. In the opening shot, an older man sitting in a garden reading fades to black by means of a black strip in the centre of the screen gradually getting wider and wider until the screen is all black, it then cuts to a shot of the canal’s bank. In the digitised version, this presents as an interesting visual effect; however, upon viewing the original film on a manual viewer, we were able to recognise the actual creative practice involved in creating this effect. To do this, Currivan hand cut a tiny piece of either black plastic or paper into a long triangle that first takes up the whole frame and gets thinner and thinner until it comes to a very fine point and disappears. This piece of black material was then taped onto the reel, most likely with splicing tape, covering about 20 frames. The entire manipulation also had to be thin enough to allow the film to run easily through a projector or viewer. Even upon viewing the film on a manual viewer over 60 years later, the film ran through so smoothly that we almost missed this fade, our 21^st^ century eyes so accustomed to visual effects. While digitisation, importantly, offers more democratic access to archival collections of films, it remains the case that the intentionality, as well as the technical consideration and practice that goes into creating a fade like Currivan’s is somewhat lost in digitisation. Without access to the original film, Currivan’s technique here would have been left to speculation.

While *Up the Canal* also features sequences of the Currivan children, along with shots of boats and the canal in action, it challenges stereotypical ideas about the home movie, not only for the techniques discussed above, but also for its use of sound. It is very rare to find Standard 8 mm film with sound, partially because the process of adding sound to most Standard 8 mm film was fairly involved and required specific equipment. Liz Czach remarks on the contrast between 1920s Hollywood’s “wholesale irreversible conversion to sound” and the fact that “a simple and convenient sound filmmaking system eluded amateur filmmakers for decades,” and observes that ultimately no single sound technology was ever adopted en masse for amateur filmmakers (Czach 2018, 77). It wasn’t until the 1970s that Super 8 cameras appeared with the ability to record sound in-camera onto pre-striped “sound film”. Prior to that, the relatively rare and expensive Fairchild Sound 8 camera did offer synced sound for regular 8 mm, but the so-called “double-system” was still the main system available to 8 mm filmmakers who wanted to utilise sound. With the “double-system”, sound is recorded separately from the image and added afterwards, meaning the strip had to be added to the reel post-processing. Once the strip was added, the filmmaker then used an 8 mm sound projector to record their audio. A 1961 issue of *Journal of the University Film Producers Association* reveals that, as of 1961, there were a total of about six 8 mm sound projector models available (*JUFPA* 1961, 13). After Kodak introduced magnetic sound-on-film Super 8 cameras in the 1970s, there were over 70 models available by the end of 1978 (Czach 2018, 80). Though sound was less accessible when Currivan was making her films, we can see her experimenting with and successfully using the complex and time-consuming double-system on four of her films.

One might expect sound to accompany Currivan’s more cinematic films like *Up the Canal* and *A Day to Remember,* but there are two other films in the collection, Reel 15 and Reel 19 made in 1959, that also utilise sound. What is particularly interesting about the crafting of these films is the extent to which they evidence the self-training Currivan was undertaking and which, no doubt, supported her later production of *Up the Canal* and *A Day to Remember.* The first of the two, Reel 15, is reminiscent of *A Day to Remember*, which documents the First Communion of Currivan’s daughter, Helen. The first section of Reel 15 revolves around older brother Dan’s First Communion, albeit accompanied by music alone and no voiceover narration unlike *A Day to Remember*. The film then has shots of Dan’s seventh birthday, children playing in the garden, and a trip to Butlin’s in 1960, and is accompanied by either music or voiceover narration recorded by Currivan’s husband, P. J. Currivan. The film’s oscillation between music and voice over, whereby each form of sound appears consecutively rather than concurrently, suggests that Currivan was learning the technique of creating an additional soundtrack.

Reel 19 also represents an early attempt at, or perhaps an early experiment with, 8 mm sound recording by Currivan. This short film, one of the three in the collection that does not utilise titles of any kind, is just under four minutes long and is a compilation of scenes of children playing around a holiday caravan along with shots of a train. There is a brief moment at the start where music is recorded and cut off as the camera follows a car driving onto the grass with children hanging out the windows and standing on the bumper. This is followed by faraway voice that is difficult to make out and then Currivan’s voice saying, “one, two…one, two, three, four. Recording. Children leaving car” as she and lots of children get out of the car. Later, there is another attempt to add music and more faint speech, and throughout the rest of the reel, the sound of the projector whirring cuts in and out. All of this serves to emphasise the experimental nature of the sound on this reel and shows Currivan testing techniques she would later come to use quite skilfully.

These early attempts contrast greatly with the highly sophisticated sound in *Up the Canal* and *A Day to Remember,* each of which as a complex soundscape that required extensive knowledge of and skill to utilise the double-system. We know that recording sound for regular 8 mm was an uncommon and complicated practice, so to see Currivan experimenting with it, we argue, identifies her as what Tepperman would call an “advanced amateur” (Tepperman 2014). According to Tepperman, the advanced amateur stands apart from the casual home movie maker insofar as they are more likely to adopt and practice new cinematic techniques and acquire additional equipment that might help them keep up with mainstream movie making. This advanced amateurism is, we argue, especially evident in Currivan’s film reels: in the quality and quantity of splices, the unique fade effect, the animations and Currivan’s use of sound. In Young’s interview with Dan Currivan, he confirms that his mother “was not interested in a mere ‘point-and-shoot’ technique” (2014, 94) and the ways she manipulates the reels in her collections only serve to add to this impression of her.

## Conclusion

Margaret Currivan exhibits an extensive understanding of amateur film production and storytelling, employing sophisticated techniques and demonstrating accomplished filmcraft such as creative titles and intertitles, splicing techniques, handmade fade techniques, stop motion animation, and double-system Standard 8 mm sound recording. All of this becomes especially evident when one examines the physical film material. While some of the techniques are visible in the digitised copies of the films, knowledge of how the films were crafted and how these techniques were implemented only reveal themselves through the physical reels.

Currivan’s filmmaking and craft, if one only focuses on the content of the films, views the digitised copies and reads the archival metadata, might easily be underestimated. Indeed, the descriptions of the various family events, children at play, domestic rituals and ordinary relationships and interactions seem to situate the filmmaker within the ‘home mode’ defined by Chalfen (1987). Although the concept of the home mode was developed as an effort to include a broader range of filmmaking within the overarching taxonomy of amateur film, it nonetheless has had implications for how films concerned with daily and homelife are understood and, indeed, stereotyped. Drawing upon Chalfen’s concept of the home mode, for example, David Buckingham, Maria Pini and Rebekah Willett (2011, 2) suggest that home movie makers “may not care much about the quality or the aesthetic character of what they produce or about the technological potentialities of their equipment”. Scenes of domestic life are, therefore, imagined to be inevitably poor quality and low-skilled productions, but there are, of course, power and gender dynamics at play here. Whether or not the home movie maker is a man or a woman, the feminised space of the family and the home confer it an inferior status. In our rebuttal of this position, we have identified Currivan as a skilled home movie maker and craftsperson who uses film to satisfy her interest in family and her family to satisfy her interest in film. While her interest in family is evidenced in the content, we find that her interest in film is revealed as much through the film reels as the film content. However, recent turns towards digitisation for the purposes of facilitating access to archival holdings risk negating physical film’s importance as a record of amateur film craft. Our purpose in this article has been to demonstrate the historical and authorial importance of the film material as records of filmmaking technique and craft. And this, in turn, foregrounds the importance of film archives as sites where the materiality of film is emphasised and preserved.

## Data Availability

Data sharing is not applicable to this article as no new data were created or analysed in this study.

## References

[CIT0001] Antoniazzi, Luca. 2021. “Digital Preservation and the Sustainability of Film Heritage.” *Information, Communication & Society* 24 (11): 1658–1673. doi:10.1080/1369118X.2020.1716042

[CIT0002] Arnold, Sarah, and Carolann Madden. 2024. “Hidden in Plain Sight: Attending to Women’s Amateur Filmmaking Histories at the Irish Film Archive.” *Historical Journal of Film, Radio and Television* 44 (2): 300–318. doi:10.1080/01439685.2023.2296233

[CIT0003] Brennen, J. Scott, and Daniel Kreiss. 2014. “Digitalization and Digitisation.” *Culture Digitally* 8 (2): 10–19.

[CIT0004] Brickell, Katherine, and Bradley L. Garrett. 2013. “Geography, Film and Exploration: Women and Amateur Filmmaking in the Himalayas.” *Transactions of the Institute of British Geographers* 38 (1): 7–11. doi:10.1111/j.1475-5661.2012.00505.x

[CIT0005] Buckingham, David, Maria Pini, and R. Rebekah Willett. 2011. *Home Truths?: Video Production and Domestic Life*. Michigan: University of Michigan Press.

[CIT0006] Chalfen, Richard. 1987. *Snapshot Versions of Life*. Bowling Green, OH: Bowling Green State University Press.

[CIT0007] Cherchi Usai, Paolo. 1995. “Film Preservation and Scholarship.” *Film History* 7 (3): 243–244.

[CIT0008] Usai, Paolo Cherchi. 2010. “The Conservation of Moving Images.” *Studies in Conservation* 55 (4): 250–257. doi:10.1179/sic.2010.55.4.250

[CIT0009] Cherchi Usai Paolo. 2021. “Film Provenance.” In *Provenance and Early Cinema*, edited by Joanne Bernardi, Paolo Cherchi Usai, Tami Williams and Joshua Yumibe, 23. Bloomington, IN: Indiana University Press.

[CIT0010] Cuzner, Daniel. 2009. “The Hidden World of Organised Amateur Film-Making.” In *Video Cultures: Media Technology and Everyday Creativity*, edited by David Buckingham, Rebekah Willett, 191–209. London: Palgrave Macmillan UK.

[CIT0011] Czach, Liz. 2018. “The Sound of Amateur Film.” *Film History* 30 (3): 75–102.

[CIT0012] Erens, Patricia. 1986. “The Galler Home Movies: A Case Study.” *Journal of Film and Video* 38 (3/4): 15–24.

[CIT0013] Fossati, Giovanna. 2018. *From Grain to Pixel: The Archival Life of Film in Transition*, 3rd rev edn. Amsterdam: Amsterdam University Press.

[CIT0014] Frith, Paul, and Keith M. Johnston. 2020. “Beyond Place: Rethinking British Amateur Films Through Gender and Technology-Based Perspectives.” *Screen* 61 (1): 129–137. doi:10.1093/screen/hjaa008

[CIT0015] Hill, Sarah, and Keith M. Johnston. 2020. “Making Women Amateur Filmmakers Visible: Reclaiming Women’s Work Through the Film Archive.” *Women’s.*” *Women’s History Review* 29 (5): 875–889. doi:10.1080/09612025.2019.1703541

[CIT0016] Christopher, Horak J. 1998. “Out of the Attic: Archiving Amateur Film.” *Journal of Film Preservation* 56 (50): 50–53.

[CIT0017] Howe, Maija. 2014. “The Photographic Hangover: Reconsidering the Aesthetics of the Postwar 8mm Home Movie.” In *Amateur Filmmaking: The Home Movie, the Archive, the Web*, edited by Laura Rascaroli, Gwenda Young, and Barry Monahan, 39–50. London: Bloomsbury.

[CIT0018] Johnston, M. Keith. 2025. “Back into Focus: Women Filmmakers, the Amateur Trade Press and 1960s British Amateur Cinema.” *Gender & History* 37 (1): 348–364. doi:10.1111/1468-0424.12702

[CIT0019] *Journal of the University Film Producers Association,* “Eight-mm Magnetic Sound.” 1961. 13 (2): 13–15.

[CIT0020] Kerry, Matthew. 2014. “The Changing Face of the Amateur Holiday Film In Britain as Constructed by Post-War Amateur Cine World (1945–1951).” *Historical Journal of Film, Radio and Television* 34 (4): 511–527. doi:10.1080/01439685.2014.941584

[CIT0021] Kleinhans, Chuck. 1986. “My Aunt Alice’s Home Movies.” *Journal of Film and Video* 38(3/4): : 25–35.

[CIT0022] Margaret Currivan Collection: Reel 23 [Up the Canal and A Day to Remember]. C. 1960s. Filmmaker Margaret Currivan. Held at the Irish Film Institute Irish Film Archive. Unique Record Number 13303.

[CIT0023] Margaret Currivan Collection: Reel 18. 1954, 1955. Filmmaker Margaret Currivan. Held at the Irish Film Institute Irish Film Archive. Unique Record Number 13313.

[CIT0024] Margaret Currivan Collection: Reel 10. 1958, 1959. Filmmaker Margaret Currivan. Held at the Irish Film Institute Irish Film Archive. Unique Record Number 13325.

[CIT0025] Margaret Currivan Collection: Reel 15 1960. Filmmaker Margaret Currivan. Held at the Irish Film Institute Irish Film Archive. Unique Record Number 13318.

[CIT0026] Margaret Currivan Collection: Reel 19. 1954. Filmmaker Margaret Currivan. Held at the Irish Film Institute Irish Film Archive. Unique Record Number 13312.

[CIT0027] Mariani, Andrea. 2018. “The Cineguf Years: Amateur Cinema and the Shaping of a Film Avant-Garde in Fascist Italy (1934–1943.”*Film History* 30 (1): 30–57.

[CIT0028] Mörner, Cecilia. 2011. “Dealing with Domestic Films: Methodological Strategies and Pitfalls in Studies of Home Movies from the Predigital Era.” *The Moving Image* 11 (2): 22–45. doi:10.1353/mov.2011.0023

[CIT0029] Motrescu-Mayes, Annamaria, and Heather Norris Nicholson. 2018. *British Women Amateur Filmmakers: National Memories and Global Identities*. Edinburgh: Edinburgh University Press.

[CIT0030] Nicholson, Heather Norris. 2004. “At Home and Abroad with Cine Enthusiasts: Regional Amateur Filmmaking and Visualizing the Mediterranean, ca. 1928–1962.” *GeoJournal* 59 (4): 323–333. doi:10.1023/B:GEJO.0000026705.38944.5c

[CIT0031] Noordegraaf, Julia. 2015. “Crowdsourcing Television’s Past. The State of Knowledge in Digital Archives.” *TMG Journal for Media History* 14 (2): 108–120. doi:10.18146/tmg.139

[CIT0032] O’Connell, Kasandra. 2021. “Archivally Absent? Female Filmmakers in the IFI Irish Film Archive.” *Alphaville: Journal of Film and Screen Media* 20 (20): 12–27. doi:10.33178/alpha.20.02

[CIT0033] Odin, Roger. 2014. “The Amateur in Cinema, in France, Since 1990.” In *A Companion to Contemporary French Cinema*. edited by A. Fox, M. Marie, R. Moine and H. Radner, West Sussex: Wiley Blackwell.

[CIT0034] Rascaroli, Laura, Gwenda Young, and Barry Monahan, eds. 2014. *Amateur Filmmaking: The Home Movie, the Archive, the Web*. London: Bloomsbury.

[CIT0035] Ruoff, K. Jeffrey. 1991. “Home Movies of the Avant-Garde: Jonas Mekas and the New York Art World.” *Cinema Journal* 30 (3): 6–28. doi:10.2307/1224927

[CIT0036] Salazkina, Masha and Enrique Fibla-Gutierrez. 2018. “Introduction: Toward a Global History of Amateur Film Practices and Institutions.” *Film History* 30 (1): i–xxiii. doi:10.2979/filmhistory.30.1.01

[CIT0037] Shand, Ryan. 2008. “Theorizing Amateur Cinema: Limitations and Possibilities.” *The Moving Image* 8 (2): 36–60. doi:10.1353/mov.0.0017

[CIT0038] Shand, Ryan. 2014. “Memories of Hard Won Victories: Amateur Moviemaking Contests and Serious Leisure.” *Leisure Studies* 33 (5): 471–490. doi:10.1080/02614367.2013.798346

[CIT0039] Streible, Dan. 2013. “Moving Image History and the F-Word; or, ‘Digital Film’ is an Oxymoron.” *Film History: An International Journal* 25 (1–2): 227–235. doi:10.2979/filmhistory.25.1-2.227

[CIT0040] Tepperman, Charles. 2014. *Amateur Cinema: The Rise of North American Moviemaking, 1923–1960*. California: University of California Press.

[CIT0041] Tepperman, Charles. 2018. “A Recognized Screen.” The New York Annual Movie Parties from Parlor to Public. *Film History*, *30*(1), 58–85.

[CIT0042] Tepperman, Charles. 2020a. “The Amateur Movie Database.” *Screen* 61 (1): 124–128. doi:10.1093/screen/hjaa007

[CIT0043] Tepperman, Charles. 2020b. “The Complex Materiality of Amateur Cinema Research: Texts, Archives and Digital Methods Introduction.” *Screen* 61 (1): 119–123. doi:10.1093/screen/hjaa006

[CIT0044] Vinogradova, Maria. 2012. “Between the State and the Kino: Amateur Film Workshops in the Soviet Union.” *Studies in European Cinema* 8 (3): 211–225. doi:10.1386/seci.8.3.211_1

[CIT0045] Young, Gwenda. 2014. “Glimpses of a Hidden History: Exploring Irish Amateur Collections, 1930–1970.” In *Amateur Filmmaking: The Home Movie, the Archive, the Web*, edited by Laura Rascaroli, Gwenda Young, and Barry Monahan, London: Bloomsbury.

[CIT0046] Zimmermann, Patricia R. 1986. “The Amateur, the Avant-Garde, and Ideologies of Art.” *Journal of Film and Video* 38 (3/4): 63–85.

[CIT0047] Zimmermann, Patricia R. 1996. “Geographies of Desire: Cartographies of Gender, Race, Nation and Empire in Amateur Film.” *Film History* 8 (1): 85–98.

